# Synthesis, Molecular Modeling, and Biological Evaluation of Novel Tetrahydro-*β*-Carboline Hydantoin and Tetrahydro-*β*-Carboline Thiohydantoin Derivatives as Phosphodiesterase 5 Inhibitors

**DOI:** 10.1155/2011/562421

**Published:** 2011-02-23

**Authors:** Ashraf H. Abadi, Jochen Lehmann, Gary A. Piazza, Mohammad Abdel-Halim, Mohamed S. M. Ali

**Affiliations:** ^1^Department of Pharmaceutical Chemistry, Faculty of Pharmacy and Biotechnology, German University in Cairo, Cairo 11835, Egypt; ^2^Institute of Pharmacy, Department of Pharmaceutical/Medicinal Chemistry, Friedrich-Schiller University, Philosophenweg 14, 07743 Jena, Germany; ^3^Division of Drug Discovery, Department of Biochemistry and Molecular Biology, Southern Research Institute, The University of Alabama at Birmingham, 2000 Ninth Avenue South, Birmingham, AL 35205, USA

## Abstract

Two series of fused tetrahydro-*β*-carboline hydantoin and tetrahydro-*β*-carboline thiohydantoin derivatives with a pendant 2,4-dimethoxyphenyl at position 5 were synthesized, and chiral carbons at positions 5 and 11a swing from *R,R* to *R,S, S,R,* and *S,S*. The prepared analogues were evaluated for their capacity to inhibit phosphodiesterase 5 (PDE5) isozyme. The *R* absolute configuration of C-5 in the *β*-carboline hydantoin derivatives was found to be essential for the PDE5 inhibition. Chiral carbon derived from amino acid even if of the S configuration (*L*-tryptophan) may lead to equiactive or more active isomers than those derived from amino acid with the *R* configuration (*D*-tryptophan). This expands the horizon from which efficient PDE5 inhibitors can be derived and may offer an economic advantage. The thiohydantoin derivatives were less active than their hydantoin congeners.

## 1. Introduction

Cyclic nucleotide phosphodiesterases (PDEs) are a superfamily of enzymes responsible for the hydrolysis of cyclic adenosine 3′,5′-monophosphate (cAMP) and cyclic guanosine 3′,5′-monophosphate (cGMP) that are important intracellular second messengers playing a central role in regulating many relevant cell functions. These second messengers are converted to the biologically inactive monophosphates with subsequent termination of their physiological functions. Currently, the PDE system includes 11 families (PDE1-PDE11) comprising 21 different gene products [1–3]. 

The cGMP-specific phosphodiesterase 5 (PDE5) is abundant in the penile tissue, platelets, vascular, and smooth muscle tissues. This enzyme is the primary target for the development of small molecules, such as the well-known sildenafil (Viagra), vardenafil (Levitra), and tadalafil (Cialis) to treat erectile dysfunction [4].

Nitric oxide activates guanylatecyclase to convert GTP to the second messenger cGMP which, in turn, results in smooth muscle relaxation and the erectile response. cGMP is hydrolysed by PDE5 to inactive GMP. PDE5 inhibitors such as sildenafil thus act to inhibit cGMP breakdown and thereby facilitate penile erection in patients suffering from MED [5]. Recently, sildenafil was approved for the treatment of pulmonary hypertension (Revatio), and there are numerous emerging reports relating the elevation of cGMP to anticancer apoptotic actions. This further illustrates the potential of PDE5 inhibitors as therapeutic agents. 

Chemically speaking, the long-acting PDE5 inhibitor tadalafil is considered as a fused tetrahydro-*β*-carboline piperazinedione derivative with pendant 1,3-benzodioxol moiety at position 6 and a methyl substituent on the piperazinedione nitrogen, and it is of 2 chiral carbons both are of the absolute* R* configuration.

In the present work, we report the synthesis of novel tetrahydro-*β*-carboline analogues and evaluate the activity of these compounds as PDE5 inhibitors.

## 2. Chemistry

**Scheme 1:**
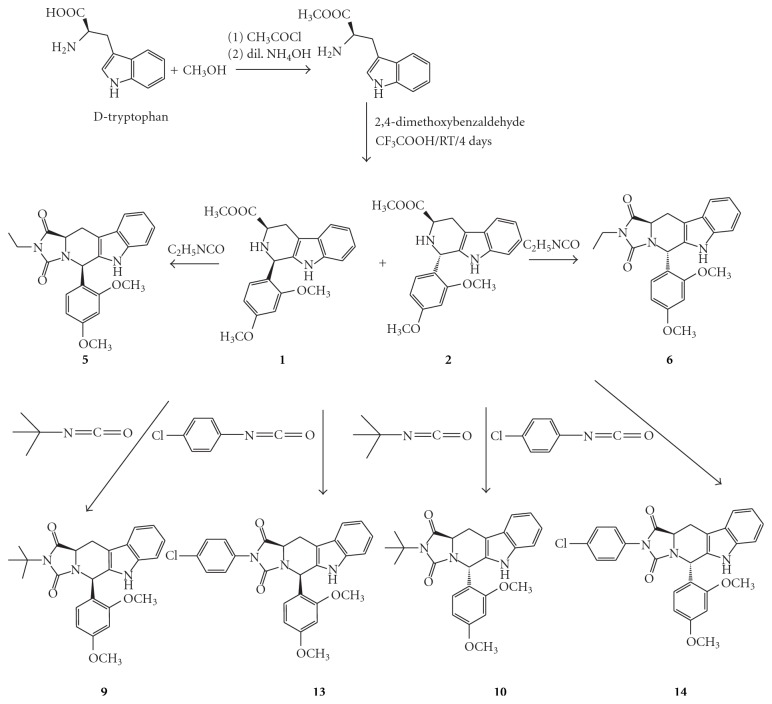
Synthesis of 1,3-disubstrituted tetrahydro-*β*-carbolines and tetrahydro-*β*-carboline hydantoin derived from *D*-tryptophan.

The general synthesis of the target *β*-carboline hydantoin and *β*-carboline thiohydantoin derivatives is illustrated in Schemes 1–4. Both *D*-tryptophan and *L*-tryptophan methyl ester were synthesized by a general synthetic procedure for amino acid esters. The ester and 2,4-dimethpxybenzaldehyde were subjected to a Pictet-Spengler reaction under nonstereospecific conditions. The diastereomeric nature of the produced *cis-* and *trans*isomers of the 1,3-disubistituted THBC (1–4) allowed their separation by column chromatography using CH_2_Cl_2_ : CH_3_OH (99.5 : 0.5) as an eluent. 

The respective pure *cis *or *trans*isomers were reacted with commercially available ethyl isocyanate, *t*-butyl isocyanate, and* p*-chlorophenyl isocyanate to produce the desired *cis- and trans*hydantoins isomers (5–16). Meanwhile, reaction with methyl and allyl isothiocyanate gave the *trans*thiohydantoins (17–20).

The assignment of *cis/trans*stereochemistry for the tetrahydro-*β*-carbolines (1–4) was based on detailed study of ^13^C-NMR spectroscopy data well established in previous literature [6]. The ^13^C-NMR signals for C-1 and C-3 are more shielded in the *trans*isomer compared to *cis*isomer, so they appear more upfield in the carbon spectrum, with Δ*δ≈*3 for C-1and Δ*δ≈*1 for C-3. This is probably due to the 1,3-diaxial spatial crowding interaction present in the *trans*isomer, with the tetrahydropyridine ring exists in half-chair conformation; the ester at C-3 is equatorially located. On cyclization to the hydantoin or the thiohydantoin derivatives proton of C-1, the same proton makes a huge downfield shift to *≈δ*6.5. This deshielding effect can be explained by the electron withdrawing effect of the recently introduced carbonyl that causes ionization of the proton attached to C-5. 

A correlation exists between *R_f_* value on TLC and the stereochemistry of the respective *β*-carboline isomer. Thus, the *cis*isomer is systematically less polar than the *trans*isomer for 1,3-disubstituted THBCs. Meanwhile, for the hydantoin series, the polarity is reversed and the *cis*isomer becomes more polar than the *trans*isomer. Thus, for compounds **1–4**, with the stereochemistry: 1*R*, 3*R*; 1*S*, 3*R*; 1*S*, 3*S,* and 1*R*, 3*S*, their *R_f_* values were 0.38, 0.19, 0.39, and 0.18; meanwhile for the corresponding hydantoins **5–8,** their *R_f_*s were 0.57, 0.68, 0.57, and 0.68, respectively, using the same elution system. 

During the attempt to synthesize the thiohydantoin series with methyl or allyl isothiocyanate, only the *trans*isomers **(17, 18)** and (**19, 20**) were obtained. The fact that treating pure *cis-*THBC with isothiocyanate would only lead to the* trans*isomer was previously discussed [7–9]. Moreover, the ^13^C-NMR, ^1^H-NMR spectra, *R_f_*, and m.p. for each couple of the thiohydantoins obtained by treating the *cis-* and* trans-*THBC derived from *D*-tryptophan with the respective isothiocyanate were completely matching with those derived from the *trans- *and *cis*-THBC isomers derived from* L*-Tryptophan, respectively. This indicates the enantiomeric nature of the two products.

Mass spectrometry to all derivatives showed the molecular ion peaks at M^+^; moreover, the THBC derivatives **1–4** showed molecular ion peak that was also the base peak indicating their stable nature. Also, compounds **1–4** showed an intense peak at M^+^-59 indicating that the Ester group (at C-3) was the most liable fragment to be lost on electron bombardment.

The infrared spectra of all derivatives showed bands at a stretching frequency around 3400 cm^−1^ for the N-H stretching. All the THBCs **1–4** showed peaks at 1750 cm^−1^ for the ester carbonyl stretching. On the other hand, the *β*-carboline-hydantoin derivatives showed 2 carbonyl stretching peaks at *≈*1760 and 1700 Cm^−1^, as one of the carbonyls is flanked between 2 nitrogen atoms, meanwhile the other is flanked between an N and a C, respectively.

## 3. Biological Results and Discussion

All the new final compounds and intermediates were evaluated for their *in vitro* ability to inhibit the recombinant human PDE5, and the potency was expressed by an IC_50_ value (50% inhibitory concentration). Most of the compounds were evaluated in 2 steps; first, the percentage inhibition at a screening dose of 10 *μ*M performed in triplicate, second, compounds displaying a percentage of inhibition >70%, the IC_50_ was determined by testing a range of 10 concentrations with at least two replicates per concentration. The results are cited in Table 1. Tadalafil was used as a positive control.

From the obtained PDE5 inhibition data, the following SAR conclusions can be withdrawn.

The THBCs **1–4** showed marginal PDE5 inhibition that seems dependent upon the stereochemistry of C-1, with compounds** 1** and **4** with C-1 of the *R* configuration more active than **2** and **3**, where C-1 is of the *S* configuration. Stereochemistry of C-5 of the *β*-carboline-hydantoin is the most crucial factor for activity. Thus, almost only those derivatives in which C-5 is of the *R* configuration (**5, 8, 9, and 12**) were the active PDE5 inhibitors. The only active compound with the C-5 S configuration was **6**; however, it is less active than its congenere with C-5 of the R configuration **5**, with IC_50_s of 0.72 versus 0.36 *μ*M. 

Interestingly, chiral carbon derived from amino acid even if with the *S* configuration (*L*-tryptophan) may lead to equiactive or more active isomers than those derived from amino acid with the *R* configuration (*D*-tryptophan). Herein, *β*-carboline-hydantoins with the C-5, C-11*a R, *and *S* configuration were more active than their analogues with the C-5, C-11*a R, *and* R* configuration this opens the horizons towards efficient PDE5 inhibitors derived from *L*-tryptophan rather than *D*-tryptophan. This may offer a highly economic advantage as* L*-tryptophan is much cheaper than *D*-tryptophan.

The size (steric) and nature of the substituent on the hydantoin nitrogen seems as a modulator for activity and relative potency. Thus, the hydantoin derivatives with C-5 of the *R*-configuration and ethyl substituent on the hydantoin N, namely **5** and **8**, both showed IC_50_s of 0.36 and 0.36 *μ*M, respectively; congener compounds but with the N*-t*-butyl substitution **9, 12 **were less active with IC_50_s of 2.4 and 0.56 *μ*M, respectively; additionally, similar compounds but with the aromatic bulkier *p*-chlorophenyl substituent were all inactive. This indicates that an aliphatic, less bulky substituent on the hydantoin N is better than bulkier aliphatic or aromatic substituent on the N. *β*-Carboline-thiohydantoins (**17–20**) are markedly less potent than the hydantoin derivatives congeners; only one congener **19** with *N*-allyl and C-5 of the *R*-configuration showed appreciable activity with IC_50_ 0.55.

It is worthy to mention that Daugan and coworkers showed that the *cis*isomer of the *β*-carboline-thiohydantoin with *N*-butyl substituent and a pendant C-5 4-methoxyphenyl or 2-methoxyphenyl were of IC_50_s of 8 nM and 1 *μ*M, respectively, versus PDE5 [10]; in our case, it seems that the 4-methoxy partly attenuates the deleterious effect of the 2-methoxy, leading to compounds with IC_50_s in between.

**Figure 1:**
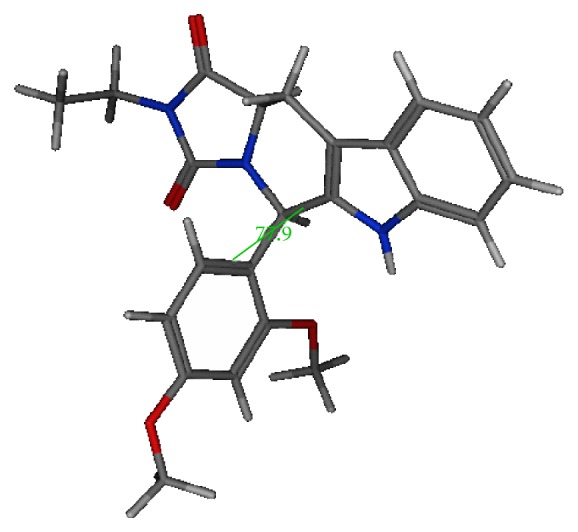
Conformational representation of the energy minimized form of compound **VIII** by MMF94 followed by conformational search, showing a semiperpendicular disposition of the pendant dimethoxyphenyl relative to the tetracyclic part. Torsional angle of 77.2°.

Interestingly, the pendant 2,4-dimethoxyphenyl showed a semiperpendicular disposition relative to the tetracycle. Interestingly, tadalafil X-ray crystal structure showed similar semiperpendicularity of the 1,3-benzodioxol relative to the tetracycle, Figure 1.

A docking experiment was implemented to dock **8 **to the human PDE5 using the MOE software [10]. For recognition between the protein and ligand, it is important that the two molecules form a stable complex. The factors contributing to the stabilization of the complex structure include complimentarily of shape, hydrogen bonding, electrostatic and hydrophobic properties, and internal strain when the complex is formed. 

**Figure 2:**
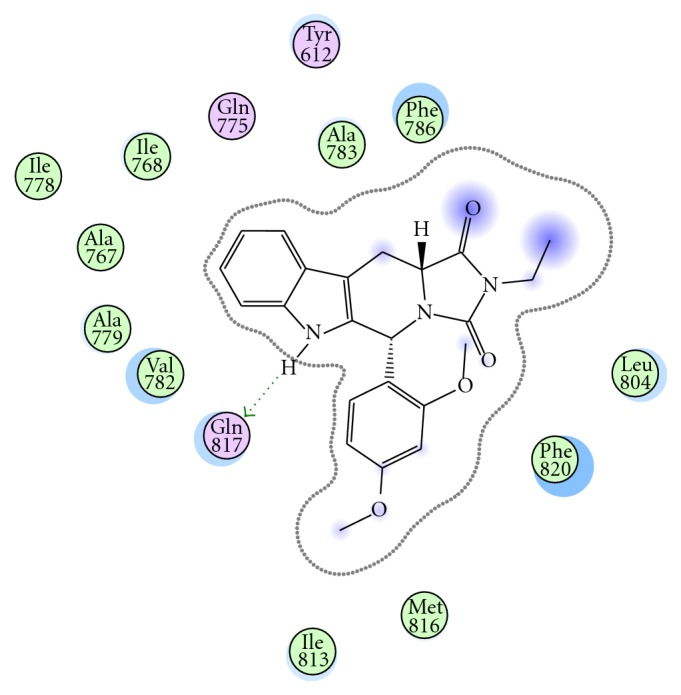
detailed photo mode showing interaction of compound VIII with human PDE5 through H-bonding with Gln 817. The missing of *π*-*π* stacking with Phe820 may be the reason why this compound is less active than tadalafil.

Detailed mode photo showed that compound **8 **is able to dock to the active pocket of PDE5 and interact with the side chain of Gln 817 which forms a single, not bidentate, hydrogen bond with the indole NH group of the respective compound; interestingly, tadalafil interacts in the same fashion; however, unlike tadalafil the *π*-*π* stacking with Phe820 is missed. This may be the reason why this compound is less active than tadalafil, Figure 2.

## 4. Experimental

### 4.1. General

All starting materials were commercially available and used without further purification. All reactions were carried out with the use of standard techniques under an inert atmosphere (N_2_). The analytical thin-layer chromatography (TLC) was carried out on E. Merck 60-F254 precoated silica gel plates, and components were usually visualized using UV light. Flash column chromatography was performed on silica gel 60 (E. Merck, 230–400 mesh). Melting points were determined on Buchi Melting Point apparatus and are uncorrected. Proton NMR (^1^H NMR) and carbon NMR (^13^C NMR) spectra were recorded at ambient temperature on Varian Mercury VX-300 MHz spectrometer using tetramethylsilane as internal standard, and proton chemical shifts are expressed in ppm in the indicated solvent. The following abbreviations are used for multiplicity of NMR signals: (s) singlet, (d) doublet, (t) triplet, (q) quadruplet, (dd) double doublet, and (m) multiplet. The elemental analyses were performed by the Microanalytical Unit, Faculty of Science, Cairo University and are within 0.4% of the theoretical value, unless stated otherwise. 

#### 4.1.1. General Procedure for the Preparation of D- and L-Tryptophan Methyl Ester

 A 250 mL round flask was charged with methanol (150 mL) and cooled with an ice water bath, then acetyl chloride (23 mL) was added dropwise using a dropping funnel over a period of 15 min. The solution was stirred for a further 10 min, then solid *D*- or *L*-tryptophan (12 g) was added in one portion, and the solution was heated to reflux for 5 hrs. The solution was allowed to cool to room temperature, and the solvent was removed under reduced pressure. The crude methyl tryptophan ester hydrochloride was extracted with ammonia solution (50 mL) and methylene chloride (5 × 50 mL). The organic layer was dried over anhydrous Na_2_SO_4_ evaporated under reduced pressure to give yellowish white oil which solidifies on cooling in almost quantitative yield. It was used without further purification.

#### 4.1.2. General Procedure for the Preparation of Methyl 1-(2,4-dimethoxyphenyl)-2,3,4,9-tetrahydro-1H-*β*-carboline-3-Carboxylate (1–4)

The appropriate tryptophan methyl ester (6.84 g, 31.4 mmol) and 2,4-dimethoxybenzaldehyde (5.73 g, 34.5 mmol) were dissolved in CH_2_Cl_2_ (25 mL) and cooled to 0°C in an ice bath. To this solution, trifluoroacetic acid (TFA) (1 mL) was added dropwise, and the mixture was stirred at room temperature for 4 days under N_2_ atmosphere. The reaction mixture was then basified with dilute NH_4_OH solution and extracted with CH_2_Cl_2_ (3 times 10 mL). The organic layer was washed with water, brine, dried over Na_2_SO_4_, filtered, and evaporated under reduced pressure. The residue was purified, and the produced diastereomers were separated by column chromatography on silica gel eluting with CH_2_Cl_2_ : CH_3_OH (99.5 : 0.5), giving first the appropriate *cis*isomer followed by the *trans *one.

**(1*R*, 3*R*) Methyl-1-(2,4-dimethoxyphenyl)-2,3,4,9-tetrahydro-1-H-pyrido[3,4-b]indole-3-carboxylate (1)**

Yield: 21%; mp 86–88°C; IR (cm^−1^) 3380 (NH), 1740(CO); 1H-NMR (CDCl_3_), *δ* ppm, 2.90–3.10 (m, 1H, CH*_a_*H*_b_*), 3.10–3.30 (m, 1H, CH*_a_*H*_b_*), 3.81(s, 3H, OCH3), 3.82 (s, 3H, OCH3), 3.87 (s, 3H, COOCH_3_), 3.9–4.0 (m, 1H, −CHCOOCH_3_), 5.7 (s, 1H, CHPh), 6.40–6.60 (m, 2H, Ar), 7.10–7.60 (m, 5H, Ar); 13C-NMR (CDCl_3_), *δ* ppm, 25.74, 51.44 (C_1_), 52.12, 55.44, 55.70, 57.04 (C_3_), 98.86, 104.88, 108.39, 110.78, 117.99, 119.40, 121.56, 125.99, 127.24, 129.92, 135.33, 135.89 160.72, 173.43. MS (m/z): 366 (M+, 100%). Anal. Calcd. for C_21_H_22_N_2_O_4_·0.5H_2_O; C: 67.17, H: 6.17, N: 7.47; Found C: 67.44, H: 5.94, N: 7.23.

**(1*S*,3*R*) Methyl-1-(2,4-dimethoxyphenyl)-2,3,4,9-tetrahydro-1-H-pyrido[3,4-b]indole-3-carboxylate (2)**

Yield: 57%; mp 146–147°C; IR (cm^−1^) 3345 (NH), 1700 (CO); ^1^H-NMR (CDCl_3_), *δ* ppm, 2.70–2.75 (m, 1H, H*_a_*H*_b_*), 2.99–3.10 (m, 1H, H*_a_*H*_b_*), 3.43–3.49 (m, 6H, OCH_3_), 3.58 (s, 3H, OCH_3_), 3.78 (m, 1H, CHCOOCH_3_), 5.42 (s, 1H, CHph), 5.95–7.74 (m, 7H, *Ar*); ^13^C-NMR (CDCl_3_), *δ* ppm, 25.08, 48.86 (*C*_1_), 51.83, 52.01, 55.32, 55.46 (*C*_3_), 98.70, 103.38, 109.03, 110.84, 117.99, 119.24, 121.62, 122.34, 126.90, 129.63, 133.10, 136.12, 157.97, 160.84, 173.90. MS (m/z): 366 (M^+^, 100%). Anal. Calcd. for C_21_H_22_N_2_O_4_·0.5H_2_O; C: 67.17, H: 6.17, N: 7.47; Found C: 67.52, H: 6.10, N: 7.35.

**(1*S,*3*S*) Methyl-1-(2,4-dimethoxyphenyl)-2,3,4,9-tetrahydro-1-H-pyrido[3,4-b]indole-3-carboxylate (3)**

Yield: 19%; mp 88–90°C; IR (cm^−1^) 3322 (NH), 1726 (CO); ^1^H-NMR (CDCl_3_), *δ* ppm, 3.02–3.07 (m, 1H, H*_a_*H*_b_*), 3.20–3.21 (m, 1H, H*_a_*H*_b_*), 3.80–3.82 (m, 9H, OCH_3_), 3.88–3.98 (m, 1H, CHCOOCH_3_), 5.6 (s,1H, CHph), 6.3–6.5 (m, 2H, *Ar*), 7.1–7.9 (m, 5H, *Ar*); ^13^C-NMR (CDCl_3_), *δ* ppm, 25.49, 51.33 (C_1_), 51.97, 55.23, 55.45, 56.70 (C3), 98.63, 104.65, 108.08, 110.73, 117.75, 119.16, 120.97, 121.36, 126.98, 129.83, 134.87, 135.79, 158.21, 160.55, 173.16. MS (m/z): 366 (M^+^, 100%). Anal. Calcd. for C_21_H_22_N_2_O_4_·0.25H_2_O; C: 68.84, H: 6.05, N: 7.65; Found C: 68.62, H: 6.09, N: 7.54.

**(1*R,*3*S*) Methyl-1-(2,4-dimethoxyphenyl)-2,3,4,9-tetrahydro-1-*H*-pyrido[3,4-b]indole-3-carboxylate (4)**

Yield: 11%; mp 148–150°C; IR (cm^−1^) 3152 (NH), 1721 (CO); ^1^H-NMR (CDCl_3_), *δ* ppm,2.94–3.02 (m, 1H, H*_a_*H*_b_*), 3.20–3.27 (m, 1H, H*_a_*H*_b_*), 3.72 (s, 3H, OCH_3_), 3.76 (s, 3H, OCH_3_), 3.82 (s, 3H, OCH_3_), 3.85–3.89 (m, 1H, CHCOOCH_3_), 5.69 (s,1H, CHph), 6.24–7.58 (m, 7H, *Ar*), 8.46 (m, 1H, NH); ^13^C-NMR (CDCl_3_), *δ* ppm, 24.94, 48.92 (C_1_), 51.55, 51.83, 55.14, 55.23 (*C*_3_), 98.51, 103.23, 108.74, 110.84, 117.76, 118.97, 121.39, 122.06, 126,71, 129.58, 132.84, 136.11, 157.79, 160.32, 173.66. MS (m/z): 366 (M^+^, 100%). Anal. Calcd. for C_21_H_22_N_2_O_4_·0.25H_2_O; C: 68.84, H: 6.05, N: 7.65, Found C: 67.79, H: 6.24, N: 7.15.

#### 4.1.3. General Procedures for the Preparation of 2-Substituted-5-(2,4-dimethoxyphenyl)-5,6,11,11a-tetrahydro-1H-imidazo[5,1-6,1]pyrido[3,4-b]indole-1,3-dione and 2-Substituted-1-oxo-5-(2,4-dimethoxyphenyl)-5,6,11,11a tetrahydro-1H-imidazo[5,1:6,1]pyrido[3,4-b]indole-3 (2H)-thione (5–16 and 17–20)

The appropriate isocyanate or isothiocyanate (1.6 mmol) was added to a well-stirred solution of the appropriate *β*-carboline (0.36 g, 1 mmol) in methyl ethyl ketone (10 mL), and the mixture was stirred at reflux for 16 hours under nitrogen atmosphere. The solvent was evaporated under reduced pressure; the residue was purified using preparative TLC and developed with CH_2_Cl_2_. The required band was stripped and dissolved in CH_2_Cl_2_. The solution was then filtered to remove the undesired silica; the filtrate was evaporated under reduced pressure to obtain the pure desired product.

***(5R,11aR)*-2-Ethyl-5-(2,4-dimethoxyphenyl)-5,6,11,11a-tetrahydro-1-H-imidazo[5,1-6,1]pyrido[3,4-b]indole-1,3-dione (5)**

Yield: 50%; mp 216–218°C; IR (cm^−1^): 3397 (NH), 1758, 1701 (CO); ^1^H-NMR (CDCl_3_), *δ* ppm: 1.22–1.28 (m, 3H,CH_2_CH_3_), 2.8–2.9 (m, 1H, H*_a_*H*_b_*), 3.49–3.55 (m, 1H, H*_a_*H*_b_*), 3.62–3.64 (m, 2H, CH_2_CH_3_), 3.79 (s, 3H, OCH_3_), 3.92 (s, 3H, OCH_3_), 4.55–4.57 (m,1H, CHCO), 6.45–6.56 (m, 3H,* Ar, and* CHph), 7.03–7.49 (m, 5H, *Ar*), 8.21 (s, 1H, NH). MS (m/z): 405 (M^+^, 100%). Anal. Calcd. for C_23_H_23_N_3_O_4_·0.8H_2_O; C: 65.80, H: 5.91, N: 10.01. Found C: 66.47, H: 5.61, N: 9.40.

**(*5S,11aR*)-2-Ethyl-5-(2,4-dimethoxyphenyl)-5,6,11,11a-tetrahydro-1-H-imidazo[5,1-6,1]pyrido[3,4-b]indole-1,3-dione (6)**

Yield: 62%; mp 190–193°C; IR (cm^−1^): 3395 (NH), 1759, 1701 (-CO-); ^1^H-NMR (CDCl_3_), *δ* ppm: 1.23–1.28 (m, 3H,CH_2_CH_3_), 2.86–2.90 (m, 1H, H*_a_*H*_b_*), 3.45–3.54 (m, 1H, H*_a_*H*_b_*), 3.62–3.65 (m, 2H, CH_2_CH_3_), 3.79 (s, 3H, OCH_3_), 3.92 (s, 3H, OCH_3_), 4.53–4.59 (m,1H, CHCO), 6.45–6.57 (m, 3H,* Ar, and* CHph), 7.03 (m, 5H, *Ar*), 8.21 (s, 1H, NH). MS (m/z): 428 (M^+^+23, 100%). Anal. Calcd. for C_23_H_23_N_3_O_4_·0.25H_2_O; C: 67.39, H: 5.78, N: 10.25. Found C: 67.45, H: 5.48, N: 9.83.

**(*5S,11aS*)-2-Ethyl-5-(2,4-dimethoxyphenyl)-5,6,11,11a-tetrahydro-1-H-imidazo[5,1-6,1]pyrido[3,4-b]indole-1,3-dione (7)**

Yield: 58%; mp 217–220°C; IR (cm^−1^) 3397 (NH), 1758, 1701 (CO); ^1^H-NMR (CDCl_3_), *δ* ppm: 1.28–1.33 (m, 3H,CH_2_CH_3_), 2.75–2.86 (m, 1H, H*_a_*H*_b_*), 3.43–3.51 (m, 1H, H*_a_*H*_b_*), 3.57–3.67 (m, 2H, CH_2_CH_3_), 3.78 (s, 3H, OCH_3_), 3.89 (s, 3H, OCH_3_), 4.51–4.57 (m,1H, CHCO), 6.43–6.55 (m, 3H,* Ar, and* CHph), 7.02–7.49 (m, 5H, *Ar*), 8.3 (s, 1H, NH). MS (m/z): 405 (M^+^, 100%). Anal. Calcd. for C_23_H_23_N_3_O_4_·0.8H_2_O; C: 65.80, H: 5.91, N: 10.01. Found C: 66.10, H: 6.06, N: 9.47.

**(*5R,11aS*)-2-Ethyl-5-(2,4-dimethoxyphenyl)-5,6,11,11a-tetrahydro-1-H-imidazo[5,1-6,1]pyrido[3,4-b]indole-1,3-dione (8)**

Yield: 87%; mp 192–195°C; IR (cm^−1^) 3397 (NH), 1759, 1701 (CO); ^1^H-NMR (CDCl_3_), *δ* ppm: 1.25–1.31 (m, 3H, CH_2_CH_3_), 2.77–2.88 (m, 1H, H*_a_*H*_b_*), 3.44–3.52 (m, 1H, H*_a_*H*_b_*), 3.58–3.67 (m, 2H, CH_2_CH_3_), 3.76 (s, 3H, OCH_3_), 3.94 (s, 3H, OCH_3_), 4.53–4.58 (m,1H, CHCO), 6.43–6.55 (m, 3H,* Ar, and* CHph), 7.02–7.52 (m, 5H, *Ar*), 8.28 (s, 1H, NH). MS (m/z): 405 (M^+^, 100%). Anal. Calcd. for C_23_H_23_N_3_O_4_·0.5H_2_O; C: 66.65, H: 5.84, N: 10.14. Found C: 66.83, H: 6.01, N: 10.39.

**(*5R,11aR*)-2-Tertiarybutyl-5-(2,4-dimethoxyphenyl)-5,6,11,11a-tetrahydro-1-H-imidazo[5,1-6,1]pyrido[3,4-b]indole-1,3-dione (9)**

Yield: 36%; mp 108–110°C; IR (cm^−1^) 3405 (NH), 1759, 1704 (CO);^1^H-NMR (CDCl_3_), *δ* ppm: 1.64 (s, 9H,C(CH_3_)_3_), 2.75–2.85 (m, 1H, H*_a_*H*_b_*), 3.43–3.49 (m, 1H, H*_a_*H*_b_*), 3.79 (s, 3H, OCH_3_), 3.91(s, 3H, OCH_3_), 4.43–4.45 (m,1H, CHCO), 6.55–7.26 (m, 8H, CHph, and *Ar*), 8.21 (s, 1H, NH). MS (m/z): 433 (M^+^, 100%), 305 (95%). Anal. Calcd. for C_25_H_27_N_3_O_4_·H_2_O; C: 66.50, H: 6.47, N: 9.31. Found C: 66:13, H: 6.51, N: 9.94.

**(5S,11aR)-2-Tertiarybutyl-5-(2,4-dimethoxyphenyl)-5,6,11,11a-tetrahydro-1-H-imidazo[5,1-6,1]pyrido[3,4-b]indole-1,3-dione (10)**

Yield: 44%; mp 191-193°C; IR (cm^−1^) 3334 (NH), 1758, 1704 (CO);^1^H-NMR (CDCl_3_), *δ* ppm: 1.65 (s, 9H,C(CH_3_)_3_, 3.41–3.59 (m, 1H, H*_a_*H*_b_*), 3.63–3.65 (m, 1H, H*_a_*H*_b_*), 3.79 (s, 3H, OCH_3_), 3.91 (s, 3H, OCH_3_), 4.40–4.47 (m,1H, CHCO), 7.04–7.52 (s, 8H, CHph, and *Ar*), 8.21 (s, 1H, NH). MS (m/z): 433 (M^+^, 91%), 305 (100%). Anal. Calcd. for C_25_H_27_N_3_O_4_·H_2_O; C: 66.50, H: 6.47, N: 9.31, Found C: 66.44, H: 6.12, N: 9.11.

**(*5S,11aS*)-2-Tertiarybutyl-5-(2,4-dimethoxyphenyl)-5,6,11,11a-tetrahydro-1-H-imidazo[5,1-6,1]pyrido[3,4-b]indole-1,3-dione (11)**

Yield: 32%; mp 103–106°C; IR (cm^−1^) 3405 (NH), 1762, 1701 (CO); ^1^H-NMR (CDCl_3_), *δ* ppm: 1.58 (s, 9H,C(C H_3_)_3_), 2.76–2.87 (m, 1H, H*_a_*H*_b_*), 3.41–3.49 (m, 1H, H*_a_*H*_b_*), 3.79 (s, 3H, OCH_3_), 3.91 (s, 3H, OCH_3_), 4.40–4.47 (m,1H, CHCO), 6.45–7.51 (m, 8H, CHph, and *Ar*), 8.21 (s, 1H, NH). MS (m/z): 433 (M^+^, 100%), 305 (82%). Anal. Calcd. for C_25_H_27_N_3_O_4_·H_2_O; C: 66.50, H: 6.47, N: 9.31. Found C: 66.23, H: 6.18, N: 9.22.

**(*5R,11aS*)-2-Tertiarybutyl-5-(2,4-dimethoxyphenyl)-5,6,11,11a-tetrahydro-1-H-imidazo[5,1-6,1]pyrido[3,4-b]indole-1,3-dione (12)**

Yield: 40%; mp 193–195°C; IR (cm^−1^) 3405 (NH), 1759, 1704 (CO); ^1^H-NMR (CDCl_3_), *δ* ppm: 1.66 (s, 9H,C(CH_3_)_3_), 3.39–3.48 (m, 1H, H*_a_*H*_b_*), 3.61–3.65(m, 1H, H*_a_*H*_b_*), 3.77 (s, 3H, OCH_3_), 3.86 (s, 3H, OCH_3_), 4.39–4.46 (m,1H, CHCO), 6.45–7.52 (s, 8H, CHph, and *Ar*), 8.35 (s, 1H, NH). MS (m/z): 433 (M^+^, 100%), 305 (74%). Anal. Calcd. for C_25_H_27_N_3_O_4_·1.25H_2_O; C: 65.85, H: 6.52, N: 9.21. Found C: 66.71, H: 6.47, N: 9.40.

**(*5R,11aR*)-2-(P-Chlorophenyl)-5-(2,4-dimethoxyphenyl)-5,6,11,11a-tetrahydro-1H-imidazo[5,1-6,1]pyrido[3,4-b]indole-1,3-dione (13)**

Yield: 53%; mp 134–136°C; IR (cm^−1^) 3373 (NH), 1773, 1714 (CO); ^1^H-NMR (CDCl_3_), *δ* ppm: 2.89–2.96 (m, 1H, H*_a_*H*_b_*), 3.52–3.61 (m, 1H, H*_a_*H*_b_*), 3.80 (s, 3H, OCH_3_), 3.88 (s, 3H, OCH_3_), 4.69–4.75 (m, 1H, CHCO), 6.45–7.49 (m, 12H, CHph, and *Ar*). MS (m/z): 487 (M^+^, 88%), 488 (M^+^+1, 25%), 305 (100%). Anal. Calcd. for C_27_H_22_ClN_3_O_4_·0.25H_2_O; C: 65.85, H: 4.61, N: 8.53. Found C: 65.56, H: 4.61, N: 8.21.

**(*5S,11aR*)-2-(P-Chlorophenyl)-5-(2,4-dimethoxyphenyl)-5,6,11,11a-tetrahydro-1-H-imidazo[5,1-6,1]pyrido[3,4-b]indole-1,3-dione (14)**

Yield: 46%; mp 214–216°C; IR (cm^−1^) 3419 (NH), 1774, 1716 (CO);^ 1^H-NMR (CDCl_3_), *δ* ppm: 2.81–2.92 (m, 1H, H*_a_*H*_b_*), 3.49–3.65 (m, 1H, H*_a_*H*_b_*), 3.79 (s, 3H, OCH_3_), 3.86 (s, 3H, OCH_3_), 4.67–4.73 (m,1H, CHCO), 6.44–7.54 (m, 12H, CHph, and Ar). MS (m/z): 510 (M^+^+Na^+1^, 100%). Anal. Calcd. for C_27_H_22_ClN_3_O_4_·0.25H_2_O; C: 65.85, H: 4.61, N: 8.53. Found C: 65.96, H: 4.76, N: 8.50.

**(*5S,11aS*)-2-(P-Chlorophenyl)-5-(2,4-dimethoxyphenyl)-5,6,11,11a-tetrahydro-1-H-imidazo[5,1-6,1]pyrido[3,4-b]indole-1,3-dione (15)**

Yield: 28%; mp 135–138°C; IR (cm^−1^) 3363 (NH), 1769, 1712 (CO); ^1^H-NMR (CDCl_3_), *δ* ppm, 2.91–3.02 (m, 1H, H*_a_*H*_b_*), 3.53–3.64 (m, 1H, H*_a_*H*_b_*), 3.80 (s, 3H, OCH_3_), 3.88(s, 3H, OCH_3_), 4.69–4.75 (m, 1H, CHCO), 6.45–7.55 (m, 12H, CHph, and Ar). MS (m/z): 487 (M+, 77%), 488 (M+ +1, 22%), 305 (100%). Anal. Calcd. for C_27_H_22_ClN_3_O_4_·0.5H_2_O; C: 65.26, H: 4.67, N: 8.46. Found C: 65.45, H: 4.78, N: 8.42.

**(*5R,11aS*)-2-(P-Chlorophenyl)-5-(2,4-dimethoxyphenyl)-5,6,11,11a-tetrahydro-1-H-imidazo[5,1-6,1]pyrido[3,4-b]indole-1,3-dione (16)**

Yield: 67%; mp 212–215°C; IR (cm^−1^) 3363 (NH), 1769, 1709 (CO); ^1^H-NMR (CDCl_3_), *δ* ppm, 2.81–2.92 (m, 1H, H*_a_*H*_b_*), 3.49–3.57 (m, 1H, H*_a_*H*_b_*), 3.79 (s, 3H, OCH_3_), 3.86 (s, 3H, OCH_3_), 4.67–4.73 (m,1H, CHCO), 6.44–7.54 (m, 12H, CHph, and *Ar*). MS (m/z): 487 (M^+^, 77%), 488 (M^+^+1, 22%), 305 (100%). Anal. Calcd. for C_27_H_22_ClN_3_O_4_·0.8H_2_O; C: 64.55, H: 4.74, N: 8.36. Found. C: 65.43, H: 4.88, N: 8.39

**(*5R,11aS*)-2-Methyl-1-oxo-5-(2,4-dimethoxyphenyl)-5,6,11,11a-tetrahydro-1H-imidazo[5,1-6,1]pyrido[3,4-b]indole-3(2H)-thione (17)**

Yield: 51%; mp 188–191°C; IR (cm^−1^) 3407 (NH), 1744 (CO), 1640 (CS); ^1^H-NMR (CDCl_3_), *δ* ppm, 2.8–2.9 (m, 1H, CH*_a_*H*_b_*), 3.53 (m, 1H, CH*_a_*H*_b_*), 3.54 (s, 3H, CH_3_), 3.79 (s, 3H,OCH_3_), 3.96 (s, 3H, OCH_3_), 4.73–4.78 (m, 1H, CHCO), 6.46–7.50 (m, 8H, CHph, and Ar), 8.19 (s, 1H, NH). MS (m/z): 407 (M^+^, 100%). Anal. Calcd. for C_22_H_21_N_3_O_3_S·0.3H_2_O; C: 57.25, H: 5.90, N: 9.10. Found C: 56.92, H: 5.52, N: 8.99.

**(*5S,11aR*)-2-Methyl-1-oxo-5-(2,4-dimethoxyphenyl)-5,6,11,11a-tetrahydro-1-H-imidazo[5,1-1,6]pyrido[3,4-b]indole-3(2H)-thione (18)**

Yield: 65%; mp 188–190°C; IR (cm^−1^) 3407 (NH), 1744 (CO), 1640 (CS); ^1^H-NMR (CDCl_3_), *δ* ppm, 2.8–2.9 (m, 1H, CH*_a_*H*_b_*), 3.5–3.6 (m, 1H, CH*_a_*H*_b_*), 3.54 (s, 3H, CH_3_), 3.79 (s, 3H, OCH_3_), 3.96 (s, 3H, OCH_3_), 4.73–4.78 (m, 1H, CHCO), 6.46–7.50 (m, 8H, CHph, and *Ar*), 8.19 (s, 1H, NH). MS (m/z): 407 (M^+^, 100%). Anal. Calcd. for C_22_H_21_N_3_O_3_S·0.3H_2_O; C: 57.25, H: 5.90, N: 9.10. Found C: 56.88, H: 5.92, N: 9.09.

**(*5R,11aS*)-2-Allyl-1-oxo-5-(2,4-dimethoxyphenyl)-5,6,11,11a-tetrahydro-1H-imidazo[5,1-1,6]pyrido[3,4-b]indole-3(2H)-thione (19)**

Yield: 58%; mp 224–227°C; IR (cm^−1^) 3415 (NH), 1725 (CO), 1613 (CS); ^1^H-NMR (CDCl_3_), *δ* ppm, 2.82–2.92 (m, 1H, CH*_a_*H*_b_*), 3.40–3.49 (m, 1H, CH*_a_*H*_b_*), 3.75 (s, 3H, OCH_3_), 3.77 (s, 3H, OCH_3_), 4.36 (m, 2H,–CH_2_–), 5.01–5.13 (m, 3H, CHCO, and CH = CH_2_), 5.76–5.87 (m, 1H, CH = CH_2_), 6.47–7.48 (m, 8H, CHph, and *Ar*). MS (m/z): 433 (M^+^+1, 100%). Anal. Calcd. for C_24_H_23_N_3_O_3_S·0.5H_2_O; C: 65.14, H: 5.47, N: 9.50. Found C: 65.25, H: 5.38, N: 9.15.

**(*5S,11aR*)-2-Allyl-1-oxo-5-(2,4-dimethoxyphenyl)-5,6,11,11a-tetrahydro-1H-imidazo[5,1-6,6,1]pyrido[3,4-b]indole-3(2H)-thione (20)**

Yield: 55%; mp 224–227°C; IR (cm^−1^) 3415 (NH), 1725 (CO), 1613 (CS); ^1^H-NMR (CDCl_3_), *δ* ppm, 2.75–2.85 (m, 1H, CH*_a_*H*_b_*), 3.45–3.54 (m, 1H, CH*_a_*H*_b_*), 3.80 (s, 3H, OCH_3_), *δ* 3.87 ppm (s, 3H, OCH_3_), 4.52–4.54 (m, 2H, –CH_2_–) 4.73–4.80 (m, 1H, CHCO), 5.21–5.33 (m, 2H, CH = CH_2_), 5.85–6.98 (m, 1H, CH = CH_2_), 6.48–7.50 (m, 8H, CHph, and *Ar*), 8.23 (s, 1H, NH). MS (m/z): 433 (M^+^+1, 100%). Anal. Calcd. for C_24_H_23_N_3_O_3_S·0.5H_2_O; C: 65.14, H: 5.47, N: 9.50. Found C: 65.71, H: 5.64, N: 9.18.

### 4.2. Biological Testing

#### 4.2.1. Phosphodiesterase Inhibitory Activity

PDE activity was measured using a modification of the IMAP fluorescence polarization phosphodiesterase assay from Molecular Devices. The assay was modified to use tetramethylrhodamine- (TAMRA-) cGMP as substrate. PDE hydrolysis of the fluorescent labeled substrate allows it to bind the IMAP binding reagent, which results in increased FP. The excitation and emission spectrum of the (TAMRA-) cGMP were at 485 and 530 nm, respectively. The assays were performed in 96-well microtiter plates using a reaction buffer containing 10 mM Tris-HCl (pH 7.2), 10 mM MgCl_2_, 0.05% NaN_3_, and 0.1% phosphate-free BSA as the carrier. Each well contained 20 *μ*L of recombinant enzyme (5 units//mL, BPS Biosciences, San Diego, CA) and 10 *μ*L test agent. The reaction was initiated by the addition of 10 *μ*L of a substrate solution containing 50 nM TAMRA-cGMP. After incubating at room temperature for 60 minutes, the reaction was terminated by adding 120 *μ*L of binding solution. FP was measured by a Perkin Elmer Envision plate reader.

**Data Analysis**

Drug effects on PDE activity were measured, and potency was expressed by an IC_50_ value (50% inhibitory concentration). The IC_50_ value was determined by testing a range of 10 concentrations with at least two replicates per concentration. Dose response curves were analyzed using Prism 4 software (GraphPad) to calculate IC_50_ values using a four-parameter logistic equation. All *in vitro* experiments involved dose-response analysis were repeated at least twice to confirm reproducibility of IC_50_ values.

### 4.3. Molecular Modeling

#### 4.3.1. Energy Minimization Procedure

The compounds with the correct stereochemistry were drawn on ChemSketch 11 and stored in mol format. The structure was recalled in molecular operating environment (MOE) [10], and all hydrogen atoms were added. The compound was energy minimized using Hamiltonian-Force Field-MMFF94x, followed by systematic conformational search (RMS gradient 0.01); the best 30 conformers were stored in an mdb database format.

#### 4.3.2. Docking

The crystal structure of human phosphodiesterase 5 complexed with tadalafil was downloaded from the protein data bank (PDB ID code 1UDU) and opened with MOE software. Only one chain out of the 2 was left for the docking experiment. Also, the old ligand was removed. The molecular operating environment of docking was used to calculate the docking energies between ligand as its conformationally searched database and the enzyme pocket as given in the software manual. The lowest energy conformation was selected as the best.

## Figures and Tables

**Scheme 2 sch2:**
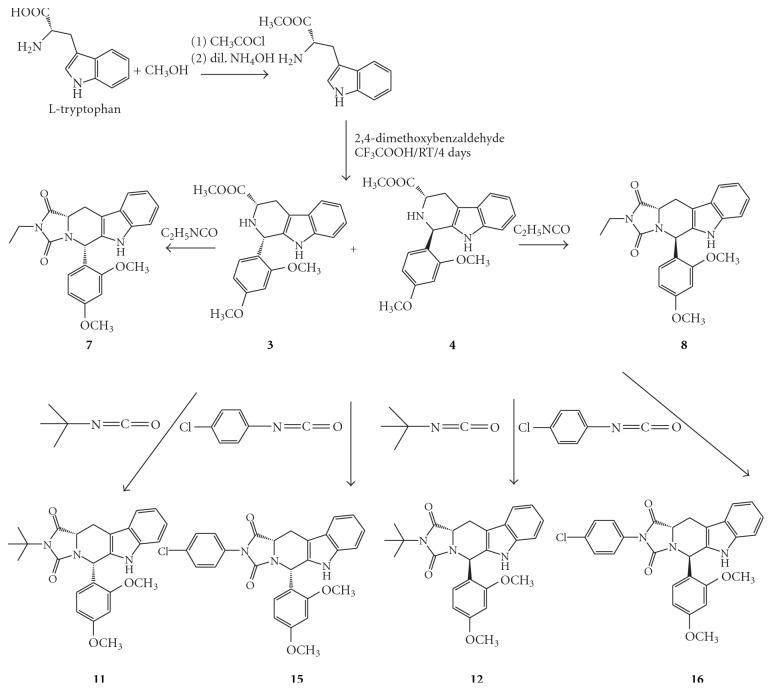
Synthesis of 1,3-disubstrituted tetrahydro-*β*-carbolines and tetrahydro-*β*-carboline hydantoin derived from *L*-tryptophan.

**Scheme 3 sch3:**
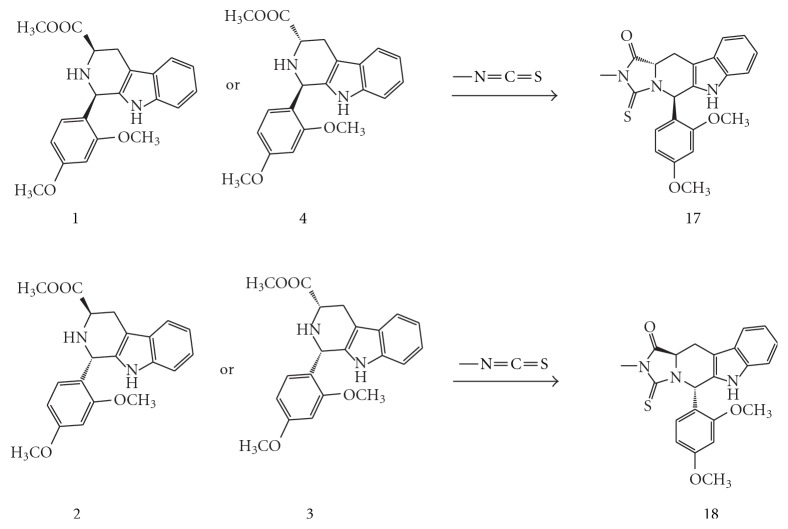
Synthesis of tetrahydro-*β*-carboline thiohydantoin derived from *D*-tryptophan.

**Scheme 4:**
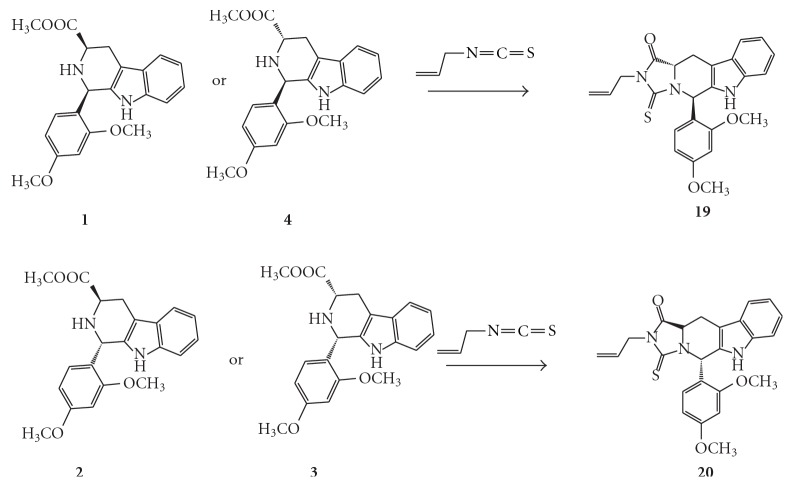
Synthesis of tetrahydro-*β*-carboline thiohydantoin derived from *L*-tryptophan.

**Table 1:** Inhibitory effect of the synthesized compounds on PDE5.

Cpd #	%PDE5 inhibition at 10 *μ*M	PDE5 inhibition IC_50_ *μ*M	Cpd #	%PDE5 inhibition at 10 *μ*M	PDE5 inhibition IC_50_ *μ*M
**1**	77	2.5	**11**	46	ND
**2**	63	ND	**12**	87	0.55
**3**	55	ND	**13**	63	ND
**4**	75	6.4	**14**	60	ND
**5**	87	0.36	**15**	35	ND
**6**	88	0.72	**16**	63	ND
**7**	54	ND	**17**	29	ND
**8**	97	0.36	**18**	23	ND
**9**	83	2.4	**19**	87	0.56
**10**	68	ND	**20**	58	ND
Tadalafil	99	0.004			
